# An improved fully-automated GMP radiosynthesis of [^18^F]fluoro-pivalic acid with solid-phase extraction purification

**DOI:** 10.1186/s41181-026-00440-4

**Published:** 2026-03-21

**Authors:** Chris Barnes, Frazer Twyman, Ramla O. Awais, Dylan Pritchard, Matthias Glaser, David R. Turton, Diana Brickute, Graham Smith, Erik Årstad, Louis Allott, Eric O. Aboagye

**Affiliations:** 1 Comprehensive Cancer Imaging Centre, Imperial College London, Hammersmith Hospital, Du Cane Road, London, W12 0NN UK; 2 Division of Radiotherapy and Imaging, The Institute of Cancer Research, 123 Old Brompton Road, London, SW7 3RP UK; 3 Positron Emission Tomography Research Centre, Faculty of Health Sciences, University of Hull, Cottingham Road, Kingston Upon Hull, HU6 7RX UK; 4 Centre for Radiopharmaceutical Chemistry, University College London, 5 Gower Place, London, WC1E 6BS UK; 5 Wolfson Brain Imaging Centre, University of Cambridge, Cambridge, UK

**Keywords:** [^18^F]FPIA, [^18^F]fluoropivalic acid, GMP radiopharmaceutical, Automated radiosynthesis, SPE purification, PET/positron emission tomography, Fluorine-18

## Abstract

**Background:**

We previously reported the first-in-human evaluation of 3-[^18^F]-fluoro-2,2-dimethylpropionic acid ([^18^F]FPIA) for imaging aberrant lipid metabolism and cancer detection. The first-generation semi-automated radiosynthesis of [^18^F]FPIA required HPLC purification to provide injection solution devoid of precursor (methyl 2,2-dimethyl-3-[(4-methylbenzenesulfonyl)oxy]propanoate), radioactive intermediate (methyl-3-([18F]fluoro)-2,2-dimethylpropanoate), and potential chemical impurities (tosic acid, 3-hydroxy-2,2-dimethylpropanoic acid and unlabelled FPIA). In readiness for global use of [^18^F]FPIA, we report a significant improvement to the GMP production through development of a fully-automated solid-phase extraction (SPE) purification method.

**Results:**

We developed a fully-automated SPE purified radiosynthesis on FASTLab™ for GMP readiness that was translated to and validated on the Trasis AIO™ platform for routine clinical use. Purification of the radiotracer by SPE on both systems was achieved (> 98% radiochemical purity), increasing the radiochemical yield compared to the HPLC-based purification method. Non-decay-corrected radiochemical yields (RCY, n.d.c) were 30.3 ± 2.3% (n = 8) and 25.8 ± 6.6% (n = 46) on GE FASTlab™ and Trasis AIO™, respectively. Non-radioactive FPIA and other analytes determined by HPLC were below the limit of detection (< 1.0 µg/mL) from GE FASTlab™ and ≤ 1.2 µg/mL from Trasis AIO™.

**Conclusions:**

The synthesis of [^18^F]FPIA was validated on the Trasis AIO™ platform for GMP production and is currently used to produce clinical doses for phase II clinical trials. Readiness for GMP validation was also demonstrated on GE FASTlab™ and can be adopted on other automated platforms.

## Background

Advances in nuclear medicine have resulted in sensitive, selective and minimally invasive diagnostic and therapeutic tools to improve cancer care (Wadsak and Mitterhauser 2010). Positron emission tomography (PET) is underpinned, among others, by radiotracers targeting tumour metabolism and provides key diagnostic, prognostic, and treatment response biomarkers in cancer (Zhu et al. 2011). Of paramount clinical importance in oncology is the imaging of glycolytic flux using 2-[^18^F]fluorodeoxyglucose ([^18^F]FDG) PET, however radiotracers targeting other metabolic pathways are being explored; fluorine-18 and carbon-11 radiolabelled acetate and choline derivatives have been investigated as imaging agents for fatty acid metabolism and membrane phospholipid metabolism, which are hallmarks of cancer (Christensen et al. 2017; Czernin et al. 2009; Leyton et al. 2009; Witney et al. 2012).

**Scheme 1:**
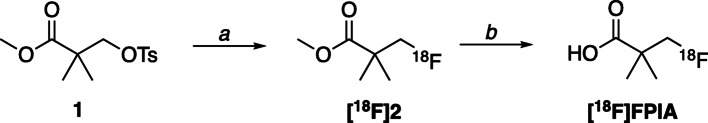
Synthesis of [^18^F]FPIA. *Reaction conditions: *a [^18^F]fluoride, K_222_, KHCO_3_, DMSO, 120 °C, 12 min; b NaOH, then HCl

We previously reported 3-[^18^F]fluoro-2,2-dimethylpropionic acid ([^18^F]F-fluoropivalate; [^18^F]FPIA), a fluorinated analogue of pivalic acid, as a radiotracer targeting early steps of the fatty acid oxidation pathway—specifically, short-chain fatty acid transcellular flux (SCFA-TF) (Pisaneschi et al. 2013). [^18^F]FPIA was synthesised in two steps from methyl 2,2-dimethyl-3-[(4-methyl benzenesulfonyl)oxy]propanoate (also known as methyl 2,2-dimethyl-3-(tosyloxy)propanoate (**1**)); the tosylate leaving group was displaced via nucleophilic substitution (S_N_2) with anhydrous [^18^F]fluoride followed by hydrolysis of the methyl ester under basic conditions (Scheme 1).

Preclinical evaluation of [^18^F]FPIA showed high in vivo uptake in breast, prostate and brain tumour models, comparable or superior to [^18^F]FDG. (Witney et al. 2014) Furthermore, [^18^F]FPIA uptake in normal brain tissue was low and therefore, unlike [^18^F]FDG, may be useful for imaging brain tumours. (Witney et al. 2014) We reported the clinical translation of [^18^F]FPIA for first-in-human (healthy volunteer) evaluation of biodistribution, safety and dosimetry (Dubash et al. 2020) and in patients with brain tumours (Islam et al. 2023) through the use of a GMP compliant semi-automated (first-generation) radiosynthesis method.

**Fig. 1:**
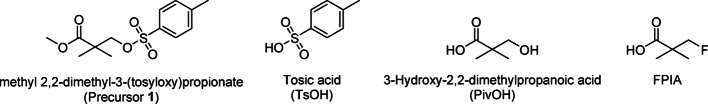
Four analytes used or produced in the radiosynthesis of [^18^F]FPIA which require elaboration in final product vial

The first-generation semi-automated procedure purified [^18^F]FPIA by semi-preparative HPLC. This was rationalised to provide injection solution devoid of precursor (methyl 2,2-dimethyl-3-[(4-methylbenzenesulfonyl)oxy]propanoate), radioactive intermediate (methyl-3-([18F]fluoro)-2,2-dimethylpropanoate), and potential chemical impurities (tosic acid, 3-hydroxy-2,2-dimethylpropanoic acid and unlabelled FPIA) (Fig. 1). A biocompatible solvent system was used to negate the requirement for a solid-phase extraction (SPE) reformulation step after the purification, thus streamlining the production. The challenge in purifying and quantifying [^18/19^F]FPIA and potential non-radioactive structural derivatives generated from the radiosynthesis, is compounded by their ionisable carboxylic acid (–COOH) moieties that required careful consideration of pH, and the lack of chromophore with UV absorption wavelengths typically used for HPLC. While the pKa of FPIA is unknown, pivalic acid is a close structural derivative with a pKa ~ 5 and is almost fully deprotonated at pH 7; therefore, all chromatographic purifications of [^18^F]FPIA, were performed using an acidic mobile phase (pH 4.5) to maintain a fully protonated species for efficient retention on a C18 solid phase. Purifying [^18^F]FPIA by C18 HPLC using a biocompatible mobile phase (ethanol:phosphate buffer pH 4.5) facilitated the prompt transfer of the radiopharmaceutical into clinical trials. Our original radiosynthesis of [^18^F]FPIA has been adapted by others for use with the GE TRACERlab FX2 N automated non-cassette-based platform, which serves niche applications in preclinical and clinical settings where the widely adopted cassette-based methodologies have not been implemented (Enriquez et al. 2026). This work demonstrates the robustness of the radiochemistry and the growing interest in establishing new preclinical and clinical studies with [^18^F]FPIA. Cassette-based radiosynthesis platforms, like the GE FASTLab™ and Trasis AIO,™ offer significant advantages to vial-based fixed reactor radiosynthesis modules, including but not limited to improved standardisation across production sites, no lengthy validated cleaning processes and routes to commercialisation for wide-scale adoption of radiopharmaceutical technology (Allott and Aboagye 2020; Bruton and Scott 2020; Shao et al. 2011). Herein, we describe a transformative advance of the radiosynthesis of [^18^F]FPIA beyond existing cassette and non-cassette based methodologies, and report a step-change in efficiency of the production through an optimised purification strategy utilising solid-phase extraction (SPE).

The quality control (QC) of the formulated dose to quantify non-radioactive FPIA was challenging due to the low UV-absorbance of the molecule, which resulted in high limits of detection (LOD) by analytical HPLC. Accurately calculating the molar activity (A_m_) of the radiotracer was problematic. The product specification for the clinical manufacture of [^18^F]FPIA included a non-radioactive FPIA limit of ≤ 30 µg in the total administered dose, which was at the limit of quantification (LOQ) by analytical HPLC (Dubash et al. 2020). Confidence in the routine production of [^18^F]FPIA would be improved by increasing LOD and LOQ, using standard detectors available in all radiopharmaceutical production facilities; as part of this work, we report a sensitive HPLC QC analysis with a significantly lower LOD and LOQ for non-radioactive impurities.

Thus, in readiness for global use of [^18^F]FPIA, we aimed to develop radiotracer production on two common commercial automated platforms (GE FASTlab™ and Trasis AIO™), only one of which was further utilised in a GMP environment (Trasis AIO™). With the first clinical productions already demonstrated on GE FASTlab™, and with the Trasis AIO™ platform available in collaborators GMP facility, we opted to conduct GMP translation on the latter system. Herein we report a significant advance to the fully-automated radiosynthesis of [^18^F]FPIA by replacing the HPLC purification with a convenient SPE method, eliminating the need for any user intervention during the synthesis.

## Methods

### Materials

Standard solvents and reagents were purchased from Sigma-Aldrich (Gillingham, United Kingdom), but specifically methanesulfonic acid for HPLC (59,510–10 × 1 mL). 2,2-Dimethyl-3-hydroxypropionic acid was purchased from Fluorochem (Hadfield, United Kingdom), absolute ethanol (product code: E/065DF/C17) was purchased from Fisher Chemical (Loughborough, United Kingdom), phosphoric acid (85–90%) for HPLC (product code: 101790675) was purchased from Fluka (Gillingham, United Kingdom) and Polyfusor Phosphates solution for infusion (product code: 23-23-761) was purchased from Fresenius Kabi (Runcorn, United Kingdom). The radiochemistry precursor (**1**) methyl 2,2-dimethyl-3-(tosyloxy)propanoate and FPIA reference standard were synthesised using a previously reported procedure (Pisaneschi et al. 2013). Sep-Pak QMA carbonate (186004051), QMA (WAT020545), C18 (WAT023501) and Oasis Prime HLB (186008886) cartridges were purchased from Waters (Elstree, Hertfordshire, UK). For developments GE FASTlab™, [^18^F]fluoride synthesised using the ^18^O(p,n)^18^F nuclear reaction was produced by Perceptive/Invicro Ltd (London, UK). Fluoride used for developments on Trasis AIO™ was produced by Wolfson Brain Imaging Centre, University of Cambridge (Cambridge, UK).

### Automated second-generation radiosynthesis of [^18^F]FPIA on the GE FASTLab™ platform

An aqueous solution of [^18^F]fluoride in oxygen-18 enriched water (2–3 mL) was transferred to the hot-cell containing the automated radiosynthesis platform. The [^18^F]fluoride was trapped on a Waters Sep-Pak QMA light cartridge and eluted into the reactor with a solution (700 µL of a 1 mL stock) containing Kryptofix 222 (9.6 mg), KHCO_3_ (1.6 mg) in MeCN:H_2_O (8:2). The [^18^F]fluoride was dried by evaporation *in vacuo* under a stream of nitrogen (80–125 °C) for 8.5 min. The reactor was cooled to 55 °C then, to the reactor containing dry [^18^F]fluoride was added precursor **1** (3.3 mg) in anhydrous DMSO (800 µL) and this mixture heated to 120 °C for 12 min to produce **[**^**18**^**F]2**. After cooling, a solution of NaOH (1.4 mL, 1 M) was added to the reactor and heated to 60 °C for 5 min to produce [^18^F]FPIA. The reactor was cooled, and the contents acidified by the addition of HCl (1.8 mL, 1 M). The reaction mixture was diluted with water (7 mL) and pushed through a Waters C18 SPE (130 mg) and Oasis Prime HLB SPE (100 mg) in series. The C18 and HLB cartridges were washed with water (15 mL) to waste. The Oasis Prime HLB SPE cartridge was eluted with a 10% ethanol (0.05% H_3_PO_4_) solution (6.5 mL) with the product solution passing through a QMA SPE (360 mg) into a product vial. This was followed by a water (3.5 mL) wash passed through the HLB and QMA cartridges to the product vial.

### Automated second-generation radiosynthesis of [^18^F]FPIA on the Trasis AIO™ platform

An aqueous solution of [^18^F]fluoride in oxygen-18 enriched water (2–3 mL diluted with water for injections (5 mL)) was transferred to the hot-cell containing the automated radiosynthesis platform. The [^18^F]fluoride was trapped on a Water Sep-Pak QMA light cartridge and eluted into the reactor with a solution (1 mL of a 10 mL stock) containing Kryptofix 222 (5 mg ± 4%), KHCO_3_ (1 mg ± 10%) in MeCN:H_2_O (9:1). The [^18^F]fluoride was dried by evaporation *in vacuo* under a stream of nitrogen (95–125 °C) for 8.25 min. The reactor was cooled to 55 °C then, to the reactor containing dry [^18^F]fluoride was added precursor **1** (3.5 mg ± 40%) in anhydrous DMSO (800 µL) and this mixture heated to 110 °C for 11.67 min to produce **[**^**18**^**F]2**. After cooling, a solution of NaOH (1.4 mL, 1.0 M) was added to the reactor and heated 50 °C for 5 min to produce [^18^F]FPIA. The reactor was cooled, and the contents acidified by the addition of HCl (1.8 mL, 1.0 M). The reaction mixture was diluted with water (7.5 mL) and pushed through a Waters C18 SPE (130 mg) and Oasis Prime HLB SPE (100 mg) in series. The reaction vessel was rinsed with water (8.5 mL) and pushed through the C18 and HLB cartridges. The C18 and HLB cartridges were washed with water (6.5 mL) and dried with N_2_ gas (8 mL) to waste. The Oasis Prime HLB SPE cartridge was eluted with a 10% ethanol (0.0383% H_3_PO_4_) solution (7.5 mL) with the product solution passing through a QMA SPE (360 mg) into a product vial. This was followed by a water (3.0 mL) wash passed through the HLB and QMA cartridges to the product vial. A solution of Phosphates solution for infusion:water (1:4.25) (3.2 mL) then water (15 mL) were added directly to the product vial.

### [^18^F]FPIA formulation for human use on Trasis AIO™

The formulation buffer was prepared as follows: A solution of diluted phosphate (solution A) was prepared using concentrated phosphate solution (10 mL, Polyfusor Phosphates Solution for Infusion, Fresenius Kabi, Cat. 23-23-761) and water for injection (WFI, 190 mL). A diluted solution of phosphoric acid (55 mL), which was obtained by adding phosphoric acid (50 µL) to WFI (100 mL) was mixed with solution A (150 mL). The pH of the mixture was adjusted to pH 6.0 and WFI added to the final volume (300 mL). The resulting solution (97.5 mL) was mixed with ethanol (2.5 mL) and passed through a non-vented sterile filter (Millex-GV, 22 µm).

### Quality control

Stock solutions of FPIA reference, precursor, and TsOH in ethanol were mixed by diluting with formulation buffer to concentrations of 1, 0.1, and 1.4 μg/mL, respectively. Spiked QC samples were obtained by mixing formulation (0.5 mL) with reference stock (50 μL) and QC sample (50 μL).

#### HPLC analysis

The HPLC equipment, thus, methodology was different in the two laboratories (different institutions) where the GE FASTlab™ and Trasis AIO™ were located. For product synthesised on the GE FASTlab™, the HPLC system used was a Shimadzu (Milton Keynes, United Kingdom) LC-10Ai Liquid Chromatograph fitted with an SPD-20A prominence UV/Vis Detector and a Phenomenex (Macclesfield, United Kingdom) Gemini 5 µm, C18 110 Å, 150 × 4.6 mm column. The UV-absorbance maxima (λ_max_) of FPIA was determined using a Nanodrop from Thermo Fisher Scientific (Cambridge, United Kingdom) in full spectrum mode.

For Trasis AIO™-based synthesis, method validation parameters including linearity, accuracy, precision, specificity, and robustness, were evaluated using the following setup. The analytical radio HPLC system was based on an Agilent 1260 Infinity II comprising of a G1311C Quat pump VL, G7116A MCT column oven, G1329B ALS autosampler, G1365D MWD VL UV detector, and a LabLogic DualScan-RAM with a 1″NaI/PMT radiodetector. The system was running Laura v6.1.5 software (LabLogic). The HPLC was run using a Phenomenex Gemini column (5 µm C18 110 Å, 150 × 4.6 mm). The mobile phase consisted of solvent A: water/0.1% MsOH (v/v), and solvent B: acetonitrile/0.1% MsOH (v/v) with the following gradient system: 0 min, B = 12%; 10 min, B = 15%; 12 min, B = 55%; 20 min, B = 55%; 21 min, B = 12%; 25 min, B = 12%. The UV absorption was recorded at 208 nm (FPIA, TsOH, unknown impurities), and 227 nm (precursor, unknown impurities). The flowrate was set to 1.0 mL/min and the injection volume to 100 µL. Molar activities of 21.2 ± 12.0 GBq/µmol were obtained.

#### Visual inspection and pH – product from Trasis AIO™

Doses were examined visually and were required to be clear, colourless and free of particulate matter. pH was confirmed using two different strips (Whatman™, Merck) across ranges 2.0–9.0 and 4.5–10. QC sample (2 µL) was placed on the centre of each window of the pH strip and colour change was compared to the manufacturers reference chart within 30 s of wetting the strip.

#### Radionuclidic identity – product from Trasis AIO™

Radionuclidic identity was confirmed by gamma-ray spectroscopy by measuring the QC sample with the multichannel analyser (RIIDEye X, Thermo Scientific) and integrating the energy peaks.

#### Bioburden, sterility and endotoxin testing – product from Trasis AIO™

Total Aerobic Microbial Count (TAMC) and Total Yeast and Mould Count (TYMC) were determined by membrane filtration, with samples processed externally (Wickham Laboratories Ltd.) Endotoxin levels were determined using the Endosafe Nextgen PTS™ system according to manufacturer guidelines. Wickham Laboratories Ltd (Hampshire, England) has demonstrated the suitability of the method for Total Aerobic Microbial Count (TAMC) and Total Yeast and Mould Count (TYMC) by Membrane Filtration procedure for one batch of [^18^F]FPIA. The testing procedure was as described in the European Pharmacopoeia Edition 10.0 section 2.6.12, the United States Pharmacopeia 43< 61 >, and the Japanese Pharmacopoeia XVII 4.05. Bioburden testing from a batch obtained from Trasis AllinOne was done using Total Viable Count by membrane filtration and Total Fungal Count by plate count. Endotoxin was tested using an Endosafe®- Nexgen PTS™ portable system (Charles River Laboratories).

#### Filter integrity testing – product from Trasis AIO™

The automated Tema Sinergie Bubble Point Tester (BPT) was used to test the integrity of Millex GV non-vented filters (SGLV033R) with a pass specification ≥ 2700 mbar.

#### Residual solvent and Kyrptofix 22.2. analysis – product from Trasis AIO™

Residual solvent analysis was determined by gas chromatography (Agilent 7890A GC System) using helium as a carrier gas at a constant flow rate of 2 mL/min through a CP-Volamine (30 m length × 0.32 mm diameter, 1.8 µm film thickness). Residual Kryptofix 222 levels were analysed using a TLC spot test whereby the intensity of QC sample (2 µL spotted into alumina TLC plate) visualised in an iodine chamber (2 min) was compared to standard solutions of Kryptofix 222 (4, 40 and 400 µg/mL) and formulation blank under the same conditions.

## Results

We developed fully-automated methodology for synthesis of [^18^F]FPIA. It should be noted that it was not our intention to compare head-to-head performance of the two systems. Instead, the approach was to first develop the SPE methodology (on GE FASTLab™) and transition that to GMP environments (the first of which is an environment that utilises Trasis AIO™). RCY, n.d.c: was 30.3 ± 2.3% (n = 8) and RCY, n.d.c: 25.8 ± 6.6% (n = 46) on GE FASTlab™ and Trasis AIO™, respectively.

### Automated solid phase extraction (SPE) method

**Fig. 2:**
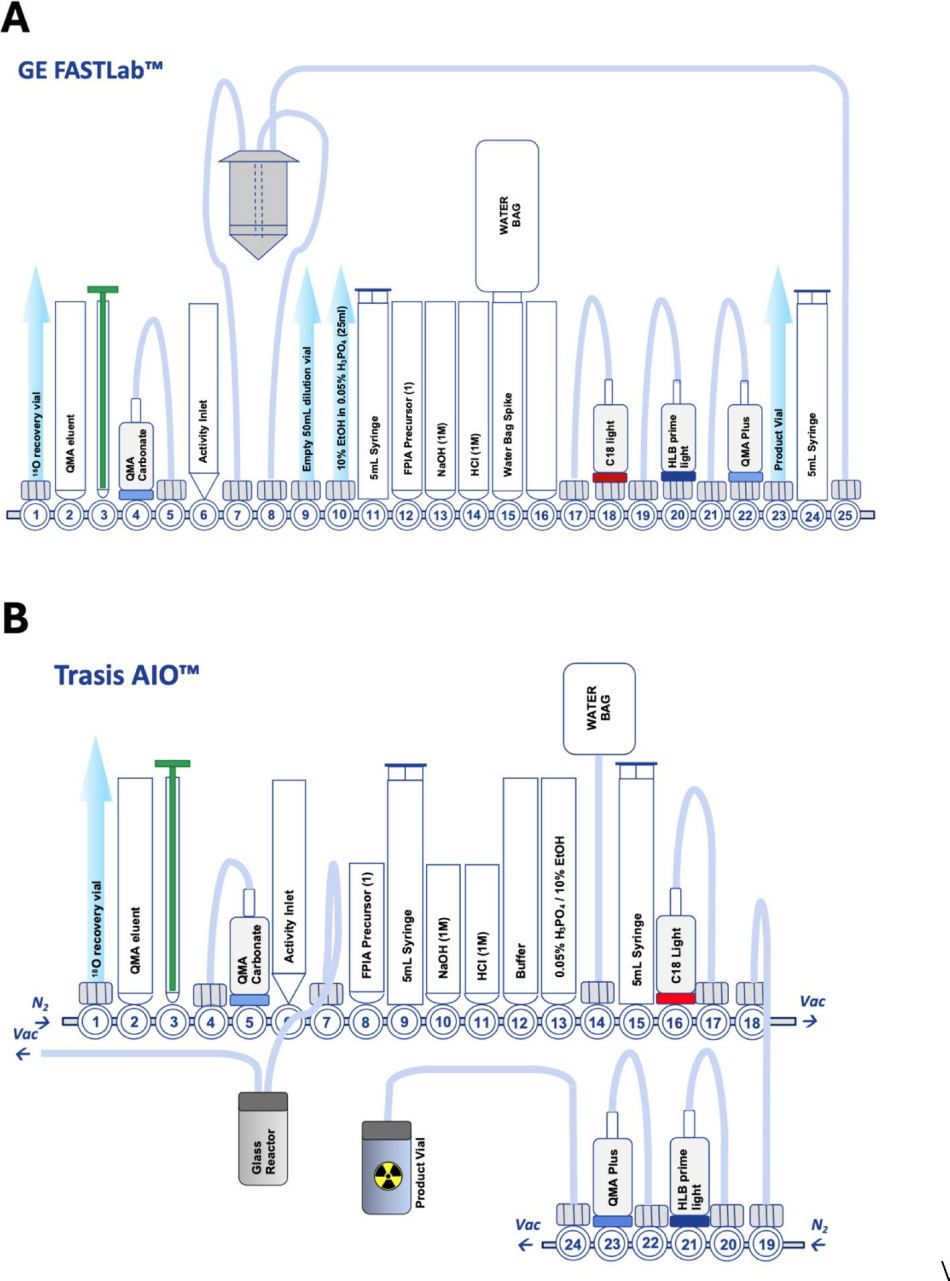
Schematic representations of **A** GE FASTLab™ cassette;** B** Trasis AIO™ cassette, with SPE cartridge-based purification

Radiosynthesis cassettes for the GE FASTlab™ and Trasis AIO™ platforms were designed and assembled as shown in Fig. 2. The improved radiosynthesis was developed on two commonly used automated platforms (GE FASTLab™ and Trasis AIO™); both platforms had sufficient space to incorporate the complete radiosynthesis and purification onto a single cassette (Table 1).

**Table 1:** Cassette reagent positions

Position	FASTLab	Trasis AIO
1	Short tube to ^18^O recovery vial	Short tube to ^18^O recovery vial / N_2_ inlet
2	Eluent vial containing 800 µL of 12 mg/mL K_222_ in MeCN and 200 µL of 8 mg/mL KHCO_3_ in H_2_O	Eluent vial containing 900 µL of 5.0 mg/mL K_222_ in MeCN and 100 µL of 10 mg/mL KHCO_3_ in H_2_O
3	Syringe 1 (1 mL)	Syringe 1 (3 mL)
4	QMA Sep-Pak Accell Plus QMA Carbonate Plus Light Cartridge	Short tube to QMA
5	Short tube to QMA	QMA Sep-Pak Accell Plus QMA Carbonate Plus Light Cartridge
6	Activity inlet reservoir	Activity inlet reservoir
7	Short tube to reaction vessel	Short tube to reaction vessel
8	Short tube to reaction vessel	Precursor vial containing 2–5 mg in 0.8 mL DMSO
9	Long line to empty 50 mL dilution vial	Syringe 2 (12 mL)
10	Long line to 50 mL vial containing 25 mL 10% Ethanol in 0.05% H_3_PO_4_	NaOH (1 M) syringe containing 1.4 mL
11	Syringe 2 (5 mL)	HCl (1 M) syringe containing 1.8 mL
12	Precursor vial containing 5 mg in 1.2 mL DMSO	Phosphates solution vial containing 2 mL
13	NaOH vial containing 2.2 mL	H_3_PO_4_ / EtOH vial (18 mL of 0.0425% H_3_PO_4_ and 2 mL EtOH)
14	HCL vial containing 2.6 mL	Waterbag (250 mL)
15	Waterbag spike	Anhydrous MeCN syringe (5 mL)
16	Empty	Sep-Pak C18 Plus Light Cartridge
17	Short tube to C18 cartridge	Short tube to C18 Plus Light SPE
18	Sep-Pak C18 Plus Light Cartridge	Long tube to bottom manifold / Vacuum
19	Short tube to HLB	Long tube to Product vial / Vacuum
20	Oasis PRiME HLB Plus Light Cartridge	Short tube to QMA Plus Short Cartridge
21	Short tube to QMA cartridge	Sep-Pak Accell Plus QMA Plus Short Cartridge
22	Sep-Pak Accell Plus QMA Plus Short Cartridge	Short tube to QMA PRiME HLB Light Cartridge
23	Long tube to product vial	Oasis PRiME HLB Plus Light Cartridge
24	Syringe 3 (5 mL)	Long tube to top manifold / N_2_ inlet
25	Long tube to reaction vessel	Not Applicable

Precursor quantity was initially optimised to reduce quantity while negligibly impacting the radiochemical yield (Table 2) using an SPE method employing C18 (130 mg), HLB (100 mg) or QMA (360 mg) cartridges. In brief, the crude reaction mixture was diluted in water and flowed through a small C18 (130 mg) and an HLB SPE (100 mg) cartridge in series. Lipophilic impurities and [^18^F]FPIA was captured on the C18 cartridge, washing with water removed the 3-hydroxy-2,2-dimethylpropionic acid impurity, most of the tosic acid and transferred [^18^F]FPIA onto the HLB SPE cartridge. The [^18^F]FPIA and remaining tosic acid was eluted from the HLB SPE cartridge with a 10% ethanol (0.05% H_3_PO_4_) solution through a final QMA SPE cartridge (360 mg); fully protonated [^18^F]FPIA passed through the QMA into the product vial containing buffer (10 mM Na_2_HPO_4_, pH 4.5–5), with residual ionised tosic acid becoming trapped on the cartridge.

**Table 2:** Precursor (**1**) quantity in relation to isolated non-decay corrected RCY GE FASTLab™ (n = 2–3, starting from 1.6 ± 0.6 GBq of [^18^F]fluoride

Precursor quantity (mg)	RCY (%)
5	31.3 ± 1.5
3	31.5 ± 3.5
1	28.0 ± 1.4

### Hydrolysis

The reaction conditions for the hydrolysis of **[**^**18**^**F]2** was also investigated. The plastic tubing inside the reactor vessel on the Trasis AIO™ platform did not withstand the basic conditions (2 M NaOH) previously used for the hydrolysis. Changing from 2 to 1 M NaOH resulted in efficient hydrolysis of **[**^**18**^**F]2** without damaging the reactor and was adopted for all further development work.

### HPLC quality control

Quality control was conducted separately on FASTLab™ and Trasis AIO™.

**Fig. 3:**
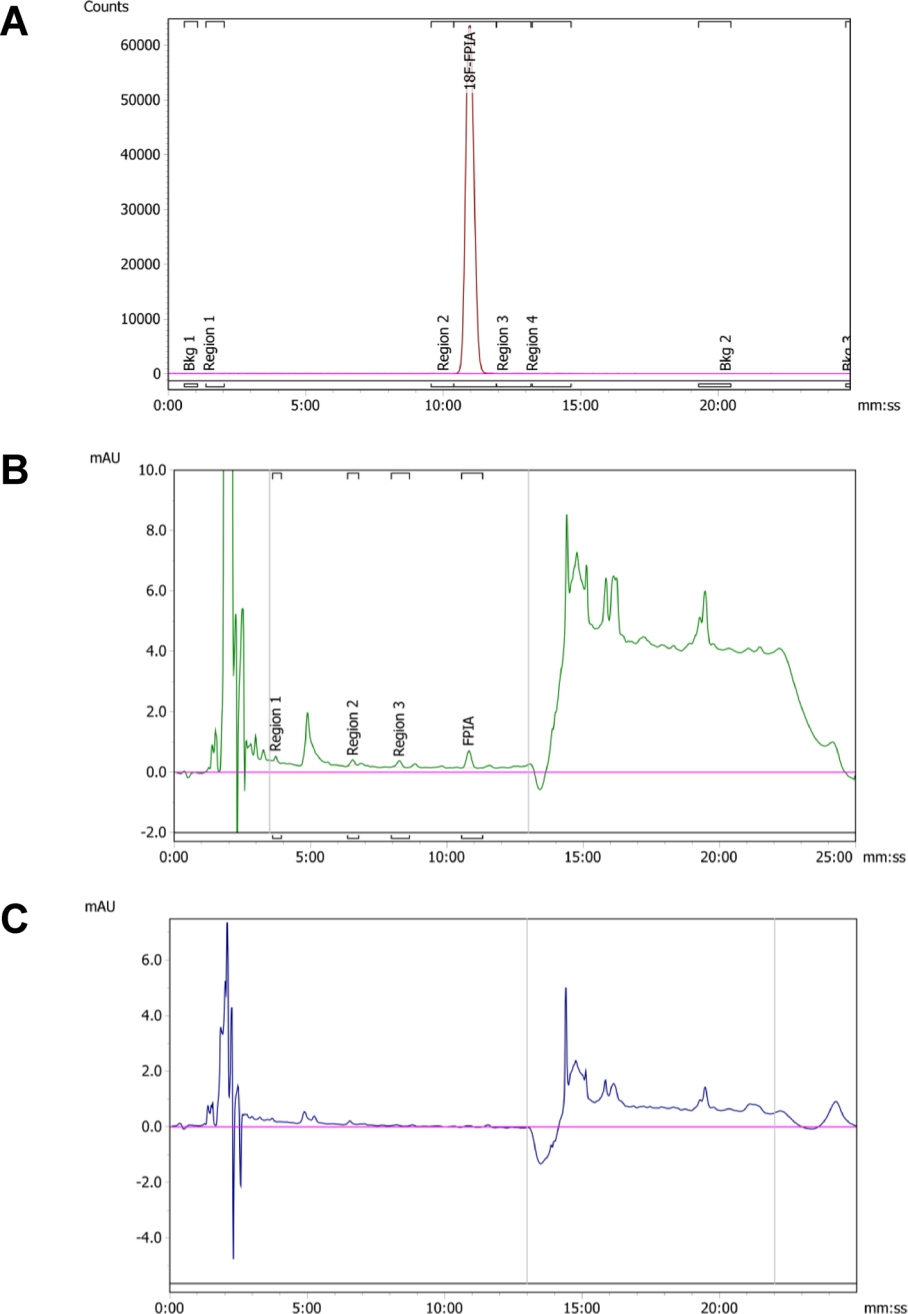
Representative chromatogram of QC sample analysis from product generated from FASTLab™ with **A** radioactivity channel (red); **B** UV detection at a wavelength of 208 nm (middle, green); **C** UV detection at a wavelength of 227 nm (bottom, blue)

**Fig. 4:**
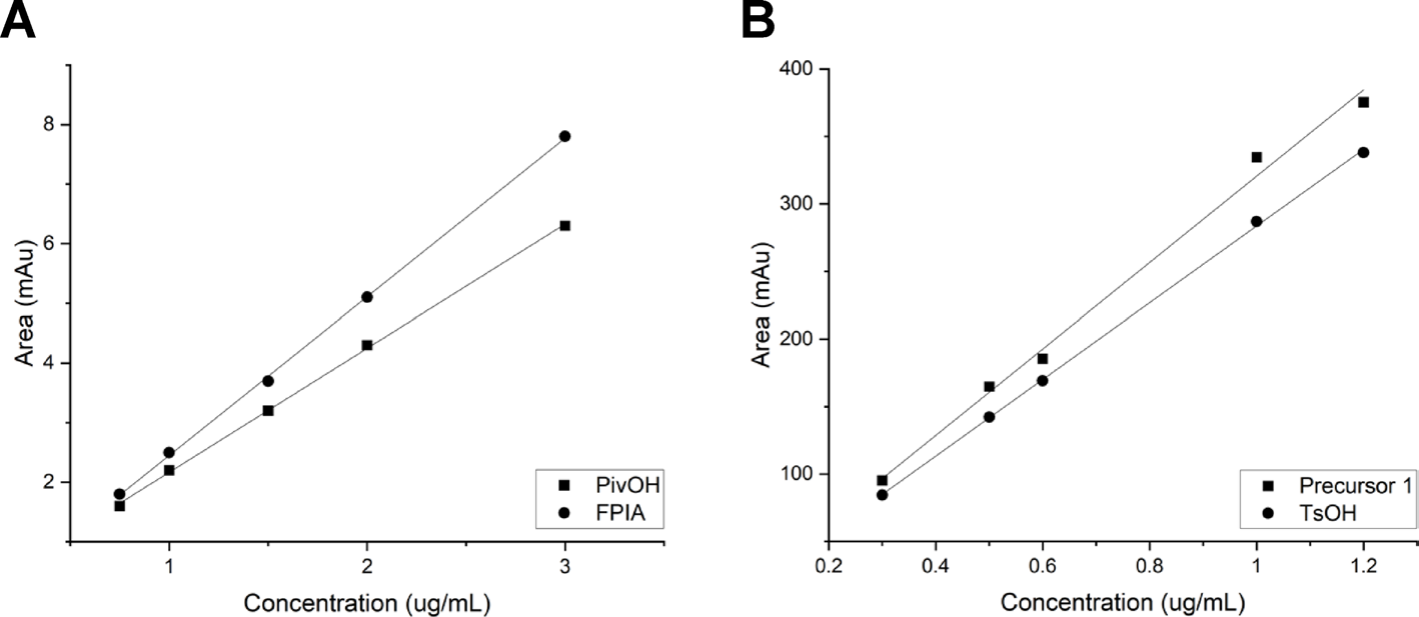
Linearity of detection by UV-HPLC in product generated from FASTLab™ for **A** low-UV absorbing analytes, 3-hydroxy-2,2-dimethylpropionic acid (PivOH) and FPIA; **B** high-UV absorbing analytes, precursor **1** and tosic acid (TsOH). All correlation coefficients (R^2^) are in the range of 0.9938–0.9996. Wavelengths of 208 and 227 nm were used for **A** and **B**, respectively, with the HPLC mobile phase (0.1% methanesulfonic acid in water and 0.2 M KCl/HCl buffer); under these conditions, low UV background absorbance was obtained

FASTLab™: HPLC QC method validation parameters including linearity, accuracy, precision, specificity, and robustness, were evaluated. The LOD of organic analytes was determined to be 0.75 μg/mL (FPIA), 0.3 μg/mL (tosic acid), 0.75 μg/mL (pivalic acid) and 0.3 μg/mL (radiochemistry precursor 1). Figure 3 shows the identity and that of detection while Fig. 4 shows linearity of approach. Recovery experiments determined the accuracy of the method. Mixed standards of known analyte concentrations were prepared and injected in triplicate. The calibration graphs were used to calculate the concentration from the linear trend equation to calculate the percentage accuracy of the experimentally determined concentration versus actual concentration. The accuracy was excellent across all analytes [FPIA 99%; PivOH 96%; precursor 1 93%; tosic acid 90%]. In summary, all analytes could be quantified down to 1 µg/mL which represents a marked improvement over the existing method (LOD ≥ 3.0 µg/mL).

Trasis AIO™: The detailed outcome of LOD and LOQ measurements within a GMP environment is shown in Table 3

**Table 3:** Summary of limit of detection (LOD) and limit of quantification (LOQ) data (µg/mL) as determined on product from Trasis AIO™ from n = 27 runs

Species	LOD^3^	LOQ^4^	LOD_MV^5^	LOQ_MV^6^
FPIA^1^	0.11	0.33	0.3	0.75
Tosic acid^1^	0.002	0.006	0.12	0.12
Hydroxy pivalic acid^1^	0.08	0.24	N/A	N/A
Precursor^2^	0.005	0.015	0.01	0.025

^1^208 nm

^2^227 nm

^3^Limit of Detection = 3 × Standard Deviation of lowest calibration concentration/Slope of Calibration Line

^4^Limit of Quantification = 3 × LOD

^5^corresponds to 10% of specified concentrations as established in HPLC Method Validation and used in QC calculations of clinical batches

^6^FPIA and Precursor: corresponds to 25% of specified concentrations, Tosic acid: corresponds to 10% of specified concentration and used in QC calculations of clinical batches

### Specification and validation data

The QC specifications generated from a total of 27 productions under GMP environment are shown in Table 4.

**Table 4:** Summary of selected QC results for clinical [^18^F]FPIA productions using the Trasis AIO™ (*n* = 27)

Test	Specification	Result
Visual identity	Clear, Colourless, Particulate Free	Passed
Sterility		Passed
pH	4.0–8.0	6.0 ± 0.3
Endotoxin	≤ 17.5 EU/mL	< 1.0 EU/mL
Kryptofix 222	≤ 50 µg/mL	< 40 µg/mL
RCP (HPLC)	≥ 95%	99.7 ± 0.3%
RCP (TLC)	≥ 95%	98.6 ± 1.1%
Stable FPIA	≤ 3.0 µg/mL	2.1 ± 1.1 µg/mL
Total Impurities	≤ 1.2 µg/mL	0.5 ± 0.8 µg/mL
Bubble Point	≥ 2700 mbar	3993 ± 197 mbar
Residual EtOH	ICH Q3C(R6)	2.3 ± 0.9%
Residual MeCN	≤ 410 µg/mL	56.9 ± 47.0 ppm
Residual IPA	≤ 5000 µg/mL	12.7 ± 8.2 ppm
Residual DMSO	≤ 5000 µg/mL	112.4 ± 95.3 ppm
TsOH		≤ 1.0 µg/mL
Precursor		≤ 0.1 µg/mL
Single Unknown Impurity		≤ 1.0 µg/mL
Total Amount of Impurities		≤ 1.0 µg/mL

## Discussion

We have developed a fully automated manufacturing of [^18^F]FPIA by SPE on two commercial platforms—GE FASTlab™ and Trasis AIO™—and further demonstrated capability of the Trasis AIO™ approach to be used for GMP production for human use. The fully automated radiosynthesis was developed to purify [^18^F]FPIA by SPE to overcome the need for any user intervention during the synthesis and improve on first generation synthesis yields associated with our original semi-preparative HPLC purification method; this would not only improve the RCY but simplify the production method. A cassette-based method which produces a radiopharmaceutical without HPLC purification is commercially attractive and simplifies national and international dissemination; (Allott and Aboagye 2020) we anticipated this to be critical for the large-scale production of [^18^F]FPIA to support multi-centre phase III trials and ultimately, routine clinical production.

An SPE purification strategy was developed and validated to efficiently isolate [^18^F]FPIA from the chemical impurities included in, or generated by, the radiosynthesis (Fig. 1). These included tosic acid (from the displacement of the tosylate leaving group), 3-hydroxy-2,2-dimethylpropionic acid and [^19^F]FPIA, appreciating challenges in pharmaceutical properties of the final product, including lack of UV chromophore and acidic nature of the analyte. In addition to removing Kryptofix 222 and solvent impurities from the final patient formulation [^18^F]FPIA was produced in an excellent radiochemical purity (RCP) > 98% as determined by HPLC and radio-TLC. Radiochemical yields were high and relatively suitable for deployment for GMP manufacturing; (RCY, n.d.c: was 30.3 and 25.8 on GE FASTlab™ and Trasis AIO™, respectively. The effect of precursor quantity on the chemical purity profile of the final formulation and the overall RCY was evaluated. The quantity of precursor (**1**) was reduced from 5 to 1 mg and the quantity of K_222_ and KHCO_3_ (9.6 mg and 2.4 mg, respectively) was reduced to 5 mg and 1 mg, respectively; reducing the quantity of reagents did not negatively impact the radiolabelling efficiency (Table 2) with precursor quantities as low as 1 mg giving excellent RCYs. Changing from 2 to 1 M NaOH avoided hydrolysis of [^18^F]2 and was adopted.

A new analytical HPLC method was developed to quantify FPIA down and other analytes included or produced by the radiosynthesis of [^18^F]FPIA. The original QC release specification for the first-in-human clinical trial stated that any amount of unknown impurity, including potentially tosic acid, should be ≤ 12 ug in the total administered dose, determined by HPLC. The amount of non-radioactive FPIA should be ≤ 30 ug in the total administered dose. The limit of detection (LOD) of the existing QC method used to quantify FPIA was ≥ 3.0 µg/mL; this work aimed to improve the sensitivity of the HPLC method while still using UV-detection. Short chain organic acids have limited UV absorbance and are often detected by ion chromatography (IC); however, specialist equipment is required (i.e. ion conductivity detection with suppression) and often, radiopharmaceutical production facilities do not have access to such facilities. It was therefore imperative, for the potential widespread use of [^18^F]FPIA in clinical studies, that an appropriate cost-effective HPLC QC method was developed that can be implemented using standard HPLC equipment fitted with a basic UV-detector.

An acidic mobile phase (ca pH 2) was required to ensure full protonation of FPIA and other organic acid impurities (e.g. 3-hydroxy-2,2-dimethylpropanoic acid, PivOH). Low UV-absorbance of the mobile phase was essential to improving the sensitivity of detection; preliminary investigations into the UV-absorbance of acidic mobile phases highlighted 0.1% methanesulfonic acid in water and 0.2 M potassium chloride/HCl, as giving low background UV absorbance at 227 nm (λ_max_ for FPIA). The methanesulfonic acid (0.1%) in water mobile phase was favoured because of its easy preparation from commercially available 1 mL ampoules and avoidance of salts which can precipitate if not fully rinsed from the HPLC system after use.

For the initial GE FASTlab™ method, a HPLC gradient developed, provided good resolution between polar and non-polar reaction components in a single chromatogram. All four analytes [PivOH (t_r_ = 3:06 mm:ss), TsOH (t_r_ = 5:58 mm:ss), FPIA (t_r_ = 8:02 mm:ss) and precursor **1** (t_r_ = 13:37 mm:ss)] were resolved within a 20 min chromatogram (Fig. 3). The HPLC method was validated for linearity of detection of all four analytes (Fig. 4). The linearity was excellent in the specified concentration range (0.75–3.0 µg/mL) [FPIA, R^2^ = 0.9996; PivOH R^2^ = 0.9995; precursor **1** R^2^ = 0.9938; tosic acid R^2^ = 0.9996]. Tables 3 and 4 shows that, the initial methodology when adopted on Trasis AIO™ was suitable for GMP use. The quantity of high and low-UV absorbing analytes produced from the first and second generation (GE FASTlab™) radiosynthesis of [^18^F]FPIA were lower than 1 µg/mL, as determined by the updated validated HPLC method. To determine the robustness of the SPE purification of [^18^F]FPIA, we used the new analytical QC method to evaluate if the quantity of precursor **1** (Table 1) influenced the UV-HPLC purity profile of the formulated [^18^F]FPIA product. No difference in UV-HPLC purity profile was observed when 1–5 mg of precursor **1** was used in the second-generation radiosynthesis, suggesting that non-radioactive analytes are effectively removed within this range.

For the Trasis AIO™, quality control of [^18^F]FPIA was carried out in accordance with the European Pharmacopoeia and could be synthesised reproducibly to meet GMP specifications (Table 4). The method has been adopted for a U.S. Food & Drug Administration authorised Investigational New Drug phase IIb trial.

As part of the process validation protocol, stability testing of the final [^18^F]FPIA was performed on three consecutive validation batches. T0 was defined as the time at which initial release testing was completed for each batch. Stability was then evaluated at T0, + 3 h, + 6 h, and + 8 h, with the product stored at controlled room temperature (19–25 °C). Temperature conditions were continuously monitored and recorded using a real-time environmental monitoring system.

All tested batches met predefined quality control specifications through the 8-h time point. Therefore, the shelf-life of [^18^F]FPIA was established as 8 h when stored at 19–25 °C.

## Conclusion

The new automated radiosynthesis of [^18^F]FPIA reported here using a fully SPE-based purification method successfully produced GMP compliant clinical doses for patients. Non-decay-corrected radiochemical yields (RCY, n.d.c) were 30.3% (n = 8) and 25.8 ± 6.6% (n = 46) on GE FASTlab™ and Trasis AIO™ platforms, respectively. Validated HPLC methods, which quantified [^19^F]FPIA and impurities to a LOD of < 1.0 µg/mL within the GE FASTlab™ and ≤ 1.2 µg/mL from Trasis AIO™ environments; a three-fold improvement on our previously reported method. The cartridge-based method may be attractive for progressing [^18^F]FPIA into routine production as the entire radiosynthesis is performed on a single cassette on both platforms in high RCY.

## Data Availability

All data have been included within the body of the manuscript. Any other information will be made available upon reasonable request to the corresponding author.

## Abbreviations

DMSO: Dimethyl sulfoxide
[^18^F]FPIA: 3-[^18^F]fluoro-2,2-dimethylpropionic acid
LOD: Limit of detection
LOQ: Limit of quantification
PivOH: Pivalic acid
RCY: Radiochemical yield
SPE: Solid phase extraction
